# Stakeholder views on the incorporation of traditional birth attendants into the formal health systems of low-and middle-income countries: a qualitative analysis of the HIFA2015 and CHILD2015 email discussion forums

**DOI:** 10.1186/1471-2393-14-118

**Published:** 2014-03-27

**Authors:** Onikepe Oluwadamilola Owolabi, Claire Glenton, Simon Lewin, Neil Pakenham-Walsh

**Affiliations:** 1 Faculty of Epidemiology and Population Health, London School of Hygiene and Tropical Medicine, Keppel Street, WC1E 7HT London, UK; 2 Norwegian Knowledge Centre for the Health Services, Oslo, Norway; 3 Health Systems Research Unit, Medical Research Council of South Africa, Tygerberg, South Africa; 4 Global Healthcare Information Network, Charlbury, Oxford, UK

**Keywords:** Traditional birth attendant, TBA, Qualitative, Community health worker, Health manpower, Social media

## Abstract

**Background:**

Health workforce shortages are key obstacles to the achievement of the health-related Millennium Development Goals. Task shifting is seen as a way to improve access to pregnancy and childbirth care. However, the role of traditional birth attendants (TBAs) within task shifting initiatives remains contested. The objective of this study was to explore stakeholder views and justifications regarding the incorporation of TBAs into formal health systems.

**Methods:**

Data were drawn from messages submitted to the HIFA2015 and CHILD2015 email discussion forums. The forums focus on the healthcare information needs of frontline health workers and citizens in low - and middle-income countries, and how these needs can be met, and also include discussion of diverse aspects of health systems. Messages about TBAs submitted between 2007-2011 were analysed thematically.

**Results:**

We identified 658 messages about TBAs from a total of 193 participants. Most participants supported the incorporation of trained TBAs into primary care systems to some degree, although their justifications for doing so varied. Participant viewpoints were influenced by the degree to which TBA involvement was seen as a long-term or short-term solution and by the tasks undertaken by TBAs.

**Conclusions:**

Many forum members indicated that they were supportive of trained TBAs being involved in the provision of pregnancy care. Members noted that TBAs were already frequently used by women and that alternative options were lacking. However, a substantial minority regarded doing so as a threat to the quality and equity of healthcare. The extent of TBA involvement needs to be context-specific and should be based on evidence on effectiveness as well as evidence on need, acceptability and feasibility.

## Background

In many Sub-Saharan African and South Asian countries, more than 50% of all births occur at home, and these countries also tend to have the highest maternal and new-born mortality rates [1,2]. Home births are most common in rural areas and amongst poorer women [3], and many of these births are attended by Traditional Birth Attendants [4,5].

**Figure 1:**
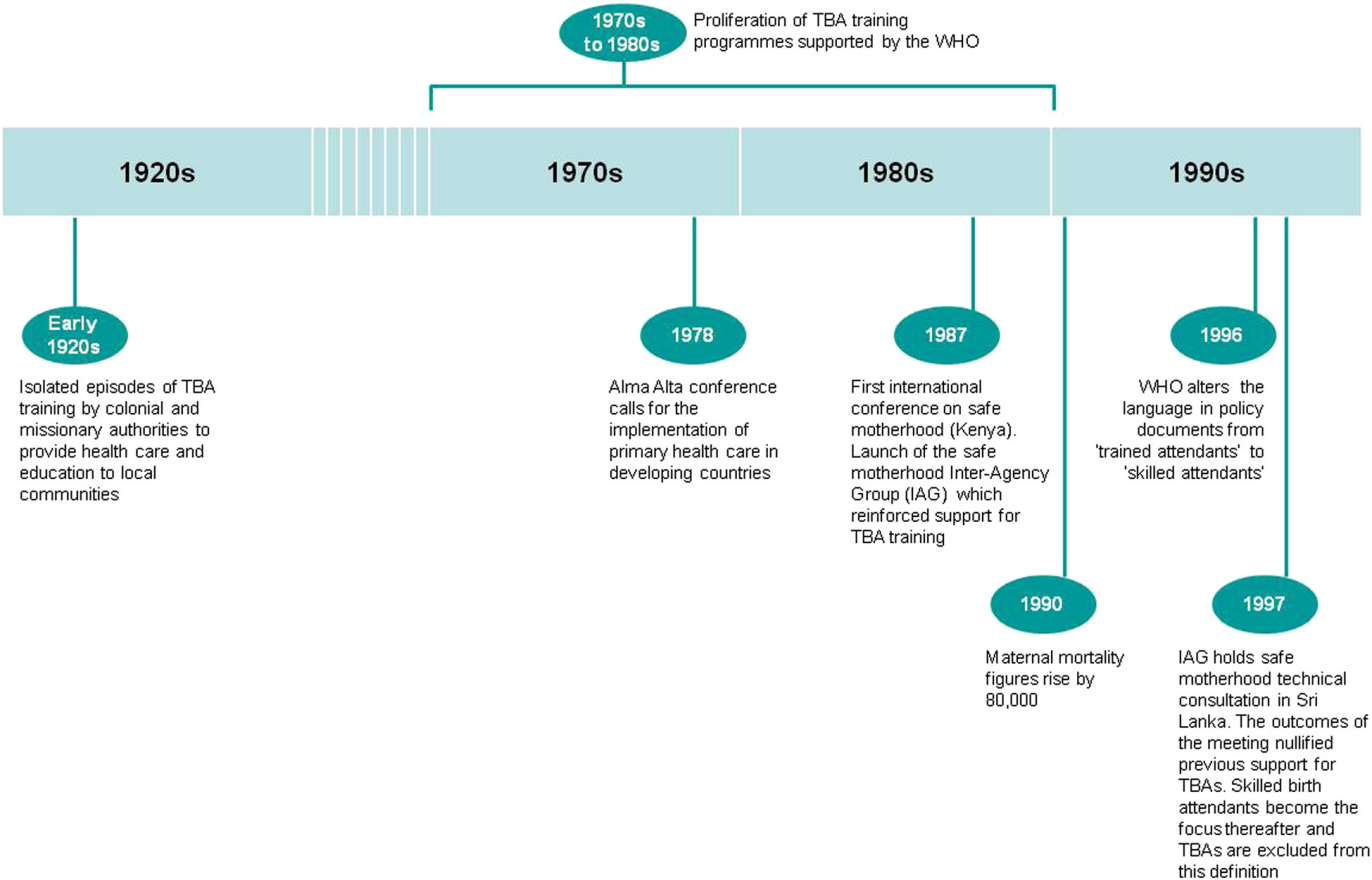
Timeline of TBA policy and training issues.

The World Health Organization (WHO) defines a Traditional Birth Attendant (TBA) as “a person who assists the mother during childbirth and who initially acquired her skills by delivering babies herself or through an apprenticeship to other TBAs” [6]. Over the last 40 years, opinions and policies on the role of TBAs have changed considerably (see Figure 1). From the 1970s until the 1990s, for example, providing TBAs with some level of biomedical training in pregnancy and childbirth was regarded as a key intervention and advocated by the WHO [7]. Although TBA training for these tasks became more widely available, estimates in 1990 indicated that maternal mortality rates had not improved [8]. At a 1997 technical consultation of the Safe Motherhood Interagency Group, TBAs were therefore formally *excluded* from the definition of skilled birth attendants [7], i.e. they were no longer included among the list of recognized health professionals (such as midwives, nurses and doctors) who had the necessary accredited skills to manage pregnancy, childbirth and postnatal care [9]. Support for TBA training was also withdrawn and a decision made to focus on the promotion and training of skilled birth attendants. A single global target was set: 90% of births would be supervised by skilled birth attendants by 2015 [10].

While discussions about the benefits and harms of trained TBAs have been extensive, evidence about the impact of TBA training on mother and child morbidity and mortality remains inconclusive. Sibley et al.’s [11] systematic review considered the effects of trained TBAs compared to untrained TBAs, but identified only one study in which this comparison was made. The study in question suggested that TBA training may reduce stillbirths and neonatal deaths, may increase referral to emergency obstetric care, and may reduce maternal deaths. However, the evidence for these outcomes was of low quality due to the risk of bias and a wide confidence interval that included both benefits and harms [11].

Just as global opinion and guidelines about TBAs have changed, so too have national policies evolved. Some countries have banned TBAs. Others, such as Malawi, have rescinded past bans amidst worsening health indicators and the challenges caused by inadequacies in the health workforce [12]. Elsewhere, in countries such as Pakistan, TBAs are officially recognized and permitted to practice [13]. Although the legal status of TBAs varies widely and differences are evident in international recommendations, TBAs are often the only childbirth support used by many women. Qualitative studies have identified a number of reasons for this ongoing use of TBAs, including poor access to health facilities, infrastructure and clinical staff; deeply rooted sociocultural practices [14]; the cost hospital care for women [15]; and the poor quality of interpersonal care in locations where healthcare is accessible [16,17]. Many women, as Gill et al. [18] reason, will continue to give birth without the supervision of a skilled birth attendant for the foreseeable future. The disjuncture between healthcare recommendations and the reality of implementing local skilled birth attendant coverage is therefore likely to persist. Policies and guidelines relating to the role of TBAs within formal health systems should therefore be revisited.

This analysis forms part of a series of studies designed to inform the World Health Organization’s ‘Recommendations for Optimizing Health Worker Roles to Improve Access to Key Maternal and New-born Health Interventions through Task Shifting’ (OPTIMIZEMNH) [19]. This OPTIMIZEMNH guidance includes a number of recommendations regarding interventions that may be delivered by lay (or community) health workers, including trained TBAs. The current study analyses contributions made to the HIFA2015 and CHILD2015 email discussion forums regarding task shifting in the context of trained TBAs, and the degree to which the TBA cadre should be incorporated in formal health systems.

### Objectives

To explore stakeholder views and justifications about the incorporation of TBAs into formal health systems.

## Methods

The HIFA2015 and CHILD2015 forums are two interdisciplinary email discussion platforms focused on the healthcare information needs of frontline healthcare workers and citizens in low-and middle-income countries (LMICs), ways to meet these needs and child health rights. In practice, discussions on the forums are far wider than their formal scope (See Table 1).

**Table 1:** The HIFA2015 and CHILD2015 forums and the TBA discussions – background information

HIFA2015 was launched in Kenya in 2006 and explores the healthcare information needs of providers in low- and middle-income countries and how these can be met. The purpose of CHILD2015 is similar, but the campaign focuses more on healthcare for children and on the health rights of children. Members of HIFA2015 and CHILD2015 interact on email discussion forums, and together they have a combined membership of more than 8,000 people based in a total of 170 countries. These forums are supported by the Dgroups Foundation, a partnership of 18 international development organizations (http://www.dgroups.info).	This wide membership greatly influences the discussion topics on the forums – these topics often extend beyond the forums’ formal remit of “information needs” to include diverse aspects of health such as the delivery of healthcare in various contexts, medical education, human resources for health and mobile health.
	The forums use a process known as Reader-Focused Moderation and this has been described in detail elsewhere [20]. This approach creates a vibrant, dynamic atmosphere, and allows topics to emerge which are of interest to the forum members.
Forum membership is diverse and includes healthcare providers, clinical students, librarians, academics, policymakers, publishers, researchers, information technologists, social scientists and other professionals from 170 countries. The organizations represented range from Ministries of Health to village health committees, from universities to primary schools, as well as intergovernmental organizations and international and local NGOs, and private and public sector providers.	The TBA topics were started mainly by the members themselves. The discussion was episodic: each episode (or topic thread) included up to 50 messages and grew over a period of weeks. Forum messages are archived automatically at http://www.dgroups.org/hifa2015 and http://www.dgroups.org/groups/child2015. However, the search and retrieval process from the archive is cumbersome and the researchers used a parallel archive instead which was created by the moderator and kept in a separate email folder.

Two researchers (OO and NPW) searched the HIFA2015 and CHILD2015 email archives independently using the free-text search terms ‘TBA’ and ‘traditional birth’. The material searched was generated from 1 January 2007 to 16 November 2011. All messages containing participant views about experiences related to TBAs were included in the study. One researcher (OO) led the thematic analysis of the data and input was provided by the other authors. Each contribution was read and re-read and the participant views and experiences were sorted into themes and categories until no new themes were discerned [21]. The definitions and boundaries of the emerging themes were discussed and agreed upon by all the authors and each contribution was then coded accordingly. Finally, data were re-arranged according to the coding scheme, summarized, and presented. The project Steering Group provided guidance on the implementation of the study.

### Ethical considerations

All HIFA2015 and CHILD2015 forum content is public. Informed consent was therefore not obtained from individual contributors, but a message was sent to members of both forums separately describing the study before it began. Those members who replied indicated that they were happy for their messages to be analysed and no concerns were expressed. All the postings were anonymized before the analysis was undertaken and none of the participant names are associated with the message extracts.

## Results

### Study participants

The discussions about TBAs were the most active in the history of HIFA2015 and CHILD2015–we included and analysed 658 messages posted by 193 participants. Although it was not always clear whether participants were referring to trained or untrained TBAs, the context and content of the messages indicated that the majority were about trained TBAs. Most of the participants were based in the WHO African region (51.8%), ~17% came from the Americas and ~20% from Europe. East Africa was the subregion with the highest number of participants (25%), while Nigerians (West Africa) posted the largest number of messages (see Tables 2 and 3 for participant characteristics).

**Table 2:** WHO regional classification of participants (country of origin/primary residence) in the HIFA2015 TBA messages analyzed

**Geographical region of participants**	**Number (%) of participants from this region**
African Region	100 (51.8%)
*Central Africa*	3 (1.6%)
*East Africa*	49 (25.4%)
*North Africa*	1 (0.5%)
*Southern Africa*	10 (5.2%)
*West Africa*	37 (19.2%)
American Region	32 (16.6%)
European Region	39 (20.2%)
Eastern Mediterranean Region	4 (2.1%)
South East Asian Region	10 (5.2%)
Western Pacific Region	8 (4.1%)
**Total**	**193 (100%)**

Participants were classified according to the WHO’s regional groupings. The following countries were represented:

African Region:

• Central Africa: Cameroon, Democratic Republic of Congo.

• East Africa: Eritrea, Kenya, Malawi, Mauritius, Mozambique, Rwanda, Tanzania, and Uganda.

• North Africa: Morocco.

• Southern Africa: Lesotho, Namibia, South Africa, and Zambia.

• West Africa: Burkina Faso, Ghana, Guinea Bissau, Nigeria, and Sierra Leone.

American Region: Canada, Haiti, St Kitts and Nevis, and USA.

European Region: Belgium, Israel, Netherlands, Norway, Portugal, Romania, Sweden, Switzerland, and the United Kingdom.

Eastern Mediterranean Region: West Bank and Gaza Strip, Pakistan, Saudi Arabia, Sudan.

South East Asian Region: India, Nepal, and Sri Lanka.

Western Pacific Region: Australia, Brunei, Fiji, Papua New Guinea and the Philippines.

**Table 3:** Professional background of participants that contributed to the TBA messages from analyzed from HIFA2015 and CHILD2015

Professional background*	**Number (%) of participants with this background**
Healthcare providers	88 (45.6%)
Programme managers	51 (26.4%)
Information providers	21 (10.9%)
Health systems	11 (5.7%)
Researchers	21 (10.9%)
Missing	1 (0.5%)
**Total**	**193 (100**%**)**

*Classification of professional background based on the most recent professional roles listed in the HIFA2015 and CHILD2015 membership profiles. These are written by the members themselves and attached to each posted message.

### Participant views on the use of TBAs in formal health systems

Participant views on the incorporation of TBAs into formal healthcare systems varied. Some suggested banning TBAs altogether but most were comfortable with working with TBAs to some degree. As we discuss below, participants justified their views with reference to pragmatic considerations (such as the lack of other options for the provision of care for women during childbirth), the continued use of TBAs by women, the willingness of TBAs to incorporate allopathic medicine into traditional processes, equity issues, and the effectiveness and safety of using trained TBAs. Participant viewpoints were influenced by the degree to which the use of TBAs was seen as a long-term or short-term solution and by the type of tasks that TBAs were expected to deliver. While there was widespread support for the use of TBAs in health promotion activities, forum members disagreed about the extent to which TBAs ought to be allowed to participate in the provision of intrapartum care.

#### Pragmatic reasons for the use of TBAs

Participants who defended the use of TBAs argued that it was better to involve and train TBAs to deliver babies than to leave women to fend for themselves. They related this to the scarcity of skilled birth attendants and lack of affordable health services. Many noted that TBAs are often the *only* available health workers in geographically inaccessible areas and usually live within or close to the communities in which they work. Using TBAs in such settings is therefore a pragmatic choice:

“In communities where the supply of formal Western trained health practitioners is scarce, one has to use what he (or she) has on the ground. In communities where there are plenty of Western trained doctors and nurses, the extent of [the] need for TBAs will be less. Also in very rural areas, where there is scarce [sic] formally trained health professionals, one cannot help but use them.” (Medical doctor, Nigeria)

#### The continued use of TBAs by women

Participants also noted that women continue to use the services of TBAs even when they have access to other formal health services. Three main reasons for this were identified:

##### Cultural appropriateness of TBAs

Many participants attributed the preference of women for TBA services to the close ties the TBAs had to the communities in which these women lived, and the knowledge and understanding that TBAs had of important local cultural practices. Some of the traditional practices provided by TBAs are not necessarily harmful and may be an important part of the social processes related to pregnancy within communities.

“It is because of … their strict adherence to faith and tradition that traditional healers are still so well patronized compared to orthodox caregivers who do the right thing by basing their decisions on scientific knowledge.” (Medical doctor, Nigeria)

##### Interpersonal relations with TBAs

Participants also noted that the close ties TBAs had to the communities in which they worked helped to foster interpersonal relations and that this may make TBAs more appealing to women:

“The relationship [with the TBA], according to the clients, is cordial. They say when you go there the TBA greet[s] you warmly and ask[s] after your family members. And by the time they asked [about] anyone you are not on good terms with, the client may break down but has a shoulder to cry on as the TBA is ready to counsel and mediate.” (NGO director, Nigeria)

##### Flexible payment mechanisms

Forum members described how TBAs were often flexible about the timing of payments from clients who could not afford to pay immediately. Payments, it was noted were typically made in cash but, in some instances, goods were given instead. This flexibility helped to ease the financial pressures faced by rural households:

“*If a pregnant woman cannot afford all charges she would be released and a visit would be paid to collect the balance later. Some, according to the TBAs, don’t pay their balance until another baby is about to be delivered.” (NGO director, Nigeria)*

This willingness to delay payment was seen as an important factor shaping client decisions about the use of TBA services, even when the fees exceeded the cost of local hospitals.

#### The willingness of TBAs to incorporate allopathic (Western) medicine into traditional processes

Some forum members noted that, in addition to TBAs using sociocultural practices that were more acceptable to women, some also incorporated elements of Western bio-medicine (practised, for example, in hospitals) and blended these with traditional medical practices:

“I have interacted with TBAs in Uganda where I come from, and I think they still need a lot of intervention though they are trying to incorporate their traditional medicine with the modern.” (Journalist, Uganda)

Participants suggested that the apparent interest of many TBAs in allopathic care may have motivated them to complete the training offered by the formal health services:

“I asked the TBAs if they would like to learn ‘Western’ medicine, and they unanimously and enthusiastically said “Yes.” The problem for them is that they didn’t meet the minimum educational requirements for being trained as formal community health workers.” (President, Private firm, USA)

Some of the forum participants, however, felt that it would be wrong to attempt to use the training of TBAs as a way to ‘convert’ them to a Western style of medical care delivery. Instead, they thought it was necessary and better to involve TBAs as healthcare providers and for the health services to commend them for utilizing the most beneficial elements of traditional healthcare models. They also felt that TBAs should be encouraged to refer any cases that were beyond their capacity. Many forum members felt that there were aspects of TBA client treatment and care that should be integrated into the hospital care system through reciprocal learning between TBAs and healthcare providers. This would help to make the care provided more culturally acceptable:

“Learning something from TBAs–in each cultural environment one could also look at what, most likely [is the] one single most destructive practice [that] TBAs might need to change and [then we could] speak about this together: TBAs and those in charge in Health Services in Districts. There might be a learning [opportunity] together instead of destroying a system that has existed much longer than the health services and that might survive health services if the trend of losing qualified staff does not change.” (NGO director, Netherlands)

#### TBAs and health service equity

Participants who argued either in favour or against the use of TBAs justified their viewpoints by referring to equity concerns, particularly related to healthcare access and healthcare quality. Some saw the utilization of TBAs as a way to plug gaps in health systems and to reduce inequities in healthcare access among vulnerable communities in resource-poor countries:

“TBAs are a major factor in health delivery, especially for pregnant women and … new-born[s]. Because the shortage of trained health manpower is most acute in the [West African] subregion, in many cases TBAs fill the gap and make the difference between ‘some care and support’ and ‘no care and death’.” (Medical doctor, Nigeria)

Others, however, argued that that the use of TBAs, even when trained, *reduced* standards of care, and contributed to the expansion of inequities in the quality of healthcare:

“This cadre will work largely in the rural areas and other underserved communities and this, of course, raises the whole question of equity and differential care for the wealthy with access to private care, and for the poor.” (Director, Private company, South Africa)

#### TBA effectiveness and safety

Participants justified their own standpoints in relation to the perceived effectiveness–or lack of effectiveness–of TBAs. Many felt that despite the many years, which had been spent on providing TBAs with delivery training and on increasing their involvement, no significant improvement in health outcomes had been achieved. Members therefore argued that TBAs should no longer be permitted to continue in this role, noting that TBAs did not take medical advice seriously or know their limits. Many TBAs, they suggested, were prone to using risky practices:

“(T)hese supporting groups (TBAs, etc.) tend to over-step their bounds and become ‘pretenders’ once they are given the basic training. They begin to pose as doctors.” (Medical doctor, Nigeria)

Other participants, however, argued that TBAs did good work among women in the community, that they saved lives, and that they were particularly effective in communicating health promotion messages:

“[TBAs] have an important role in social mobilisation and educating … pregnant women and the family in identifying danger signs early and advising them to immediately take the mother to an appropriate health facility.” (Medical doctor, Nigeria)

#### TBAs as a short-term or long-term solution

Many forum members felt that the involvement of TBAs was necessary within the health system of their country at this particular time. They argued that TBA roles should be adapted to meet the needs of specific contexts and then phased out and replaced by formally qualified health workers as soon as possible:

“Orthodox medical care is regulated by laws and malpractice is punishable. This is not the case with TBAs. Attempts should be made to regulate their practice. This should result in better practice and fewer avoidable morbidities and mortalities. The ultimate goal should be that every delivery–whether at home or institutional–should be attended by a skilled birth attendant. [The] training of skilled birth attendants should continue alongside [the] training of TBAs until … there … [are] enough skilled personnel to cater for the teeming [sic] Nigerian population of child bearing women.” (Medical doctor, Nigeria)

A few forum members felt that TBAs still had a long-term role to play within health systems and within communities:

“The TBAs are the caretakers in their villages and they can offer much wisdom in many cases that we can learn from as well. They are not a population that needs to be eradicated [sic]; they need to be embraced, educated and supported as the shortage of midwives is extreme, the women and their children need someone.” (Midwifery student, USA)

#### TBA tasks

Very few forum members talked about which *specific* tasks TBAs should or should not be allowed to perform, although implicit in many of their contributions was the notion that the use of trained TBAs was acceptable, at least for normal deliveries. Of the few participants who did discuss which particular tasks TBAs might be allowed to undertake, most indicated that they were willing to consider the involvement of TBAs in different forms of health promotion activities and suggested that TBAs should take on roles similar to lay or community health workers:

“What we could do is give them an option to train as community health workers, help them understand their role in maternal and child health [and the] dangers of unskilled deliveries. This way they [will] still feel … part of the community.” (Programme coordinator, NGO, Kenya)

Many participants in the forums also accepted that TBAs could be trained to refer women to health facilities before the onset of labour, where they would receive skilled delivery attendance:

“No matter how much training they may undergo, TBAs will not become skilled providers! They [do] however have an important role … [in] referring women to healthcare facilities where they can obtain skilled care.” (Health Advisor, Ministry of Health, Kenya)

Even fewer of the participants suggested that TBAs should be trained and permitted to assist at normal home deliveries and refer when danger signs were observed. One commentator wrote:

“They need to be supported and empowered to be able to give basic ANC [antenatal care], uncomplicated birthing services and also timely referral.” (Medical doctor, Nigeria)

The degree to which trained TBAs should be allowed to assist at deliveries was the most disputed issue among the forum contributors. One argued, for example:

“With the experience I have … [had with] TBAs, they should not be allowed to conduct deliveries … [or be] trained to do so, because they cannot get the comprehensive package about midwifery due to their limited educational background to cope with hard subjects like anatomy and physiology.” (Programme officer, Uganda)

## Discussion

The topic of whether and how to utilize TBAs within formal health systems continues to be an emotive issue among those involved in healthcare provision and management. The themes identified in our study mirror wider discourses and debates about TBAs at the national and international level and within TBA literature [22,23]. Although opinions varied among forum members, most agreed that substantial gaps were evident in their own countries with regard to facility coverage and available personnel. These views are consistent with findings from recent studies, which have shown that while skilled birth attendance coverage may be a necessary and laudable goal, global healthcare service targets are unlikely to be achieved in the short term [24,25]. Several studies have concluded that more channels of delivery will be needed if an increase in access to maternal and newborn health services is to be achieved [11,25,26]. In addition, health systems will need to be strengthened to support the delivery of key maternal health interventions, sustainably improve quality of care and reduce preventable morbidity and mortality. Forum members suggested, sometimes reluctantly, that TBAs *are* a readily available cadre which could bridge workforce gaps, at least in the short term. In addition to these practical considerations, participants noted that women continue to use TBAs even when formal health facilities are available. This, they reasoned, is because TBAs are readily available in some communities, because TBA-client relationships are often positive and incorporate shared values and beliefs, and because many TBAs demonstrate flexible attitudes regarding payment. Similar considerations have also been highlighted in other studies [2,7,15,27,28]. Health system planners should consider how such constructive elements could be integrated into the organization and planning of skilled birth attendant programmes in ways that would ensure user needs are better met.

While most participants supported at least some incorporation of trained TBAs into formal health systems, a significant minority regarded doing so as a threat to healthcare quality and equity. The notion that trained TBAs and other lay health workers provide ‘second class’ care for the poor is not uncommon [23]. Efforts to incorporate trained TBAs into the health system are likely to depend on adequate training and supervision to ensure that the quality of care they provide remains consistent. A lack of such support remains an obstacle to the success of lay health worker programmes [29]. Rigorous assessments of the impact of training on the safety and effectiveness of care delivered by TBAs are needed. We also need research about the acceptability and feasibility of using trained TBAs to conduct normal home deliveries when access to skilled birth attendants is limited.

### Study strengths and limitations

Qualitative research about TBAs has focused particularly on the viewpoints of TBAs themselves [30-34] and those of pregnant women [15,27,35]. Far fewer studies have explored the opinions of healthcare professionals regarding the incorporation of TBAs into the formal health system [28] and we were unable to identify any studies which examined the views and experiences of policymakers. Our study therefore provided an opportunity to examine the opinions of groups which have, to date, been underrepresented in the literature. Discussion arenas such as the HIFA2015 and CHILD2015 email forums enabled us to access stakeholder views and to overcome key logistical difficulties which would normally be associated with primary research involving healthcare professionals and policymakers, especially across so many countries. The geographical and cultural diversity of the forum participants has contributed to the richness of our data.

The use of data from email discussion forums had a number of advantages and disadvantages. Firstly, the data analysed in this study were not generated for research purposes. Consequently, we were unable to delve more deeply into the views of the forum participants. Secondly, we were unable to verify how representative the views expressed in the forum are of wider constituencies, and whether there are important groups whose views are entirely absent. People without access to email or the Internet, for example, would not have been able to participate in these forums. Within our study, participants from Nigeria were the most active and this may also have affected the representativeness of our findings. Thirdly, participants may have been more cautious in the ways they expressed their views within this public forum, compared to how they might have expressed their views in anonymized interviews. However, it is possible that the online format of the discussions made it easier for participants to be *more* open when talking about TBA issues. Discussing such topics in researcher-conducted face-to-face interviews, can potentially be more problematic if participants perceive a pressure to present a professional ‘front’ [36,37]. Finally, the long duration of the forum discussions allowed for different aspects of the topic to be explored in-depth and gave time for participants to express their own, evolving views [37].

## Conclusion

This study used an innovative method to explore the experiences and opinions of diverse local players regarding TBA roles within formal health systems. Our results indicate that there was support among the forum members for the involvement of trained TBAs in at least some aspects of pregnancy and childbirth. Forum members justified their support by referring to the lack of other potential options and the continued use of TBAs by women. But a substantial minority of participants regarded the involvement of TBAs as a threat both to the quality and equity of healthcare.

Decisions related to the extent to which trained TBAs should be involved in healthcare delivery need to be context-specific. The formulation of national plans should be based on global evidence on effectiveness as well as global and local evidence on need, acceptability and feasibility [19,38]. Further work is needed to strengthen this evidence base and to engage with key stakeholders. The HIFA2015 and CHILD2015 forums will continue to provide important opportunities to engage in dialogue.

## Abbreviations

LMICs: Low-and middle-income countries; OPTIMIZEMNH: Optimizing Health Worker Roles to Improve Access to Key Maternal and Newborn Health Interventions through Task Shifting; TBA: Traditional birth attendant; WHO: World Health Organization.

## Competing interests

The authors of this paper are all HIFA2015 members. Specifically, NPW was the HIFA2015 forum moderator and administrator, and the moderator of the CHILD2015 forum during the period from which the discussion data were extracted. NPW therefore contributed to the shaping of the forum discussions. It should be noted that the primary role of a forum moderator is to facilitate the sharing of experiences and perspectives among members. As such, moderators are required to be impartial and to give members the opportunity to express their own views. The role of the moderator is to approve messages for distribution by determining if these fall within the remit of the forum, to check whether the messages are intelligible, and to ensure that they do not include personal attacks on other members. All ineligible messages are referred back to the authors after the comments are submitted, and members are given an opportunity to revise and resubmit them. Moderators may express their own views on the forum through direct messages and contributions to the topics discussed. OO made one contribution to the debate about TBAs to one of the forums while CG and SL did not submit contributions on the study topic to either forum. Because the views of the authors about TBAs could potentially have influenced their own interpretations of the extracted data, we involved the other study authors during the analysis and interpretation of the data and also obtained additional feedback from the project Steering Group. The authors declare that they have no other competing interests.

## Authors’ contributions

CG, NPW and SL conceived and designed the study. All authors were involved in data extraction, analysis and interpretation, and in the writing of this manuscript. All authors read and approved the final manuscript.

## Pre-publication history

The pre-publication history for this paper can be accessed here:

http://www.biomedcentral.com/1471-2393/14/118/prepub
